# Genome-Wide Identification and Characterization of Long Non-Coding RNAs Associated with Floral Scent Formation in Jasmine (*Jasminum sambac*)

**DOI:** 10.3390/biom14010045

**Published:** 2023-12-28

**Authors:** Zhaogeng Lu, Xinwen Wang, Xinyi Lin, Salma Mostafa, Hongyan Bao, Shixiong Ren, Jiawen Cui, Biao Jin

**Affiliations:** 1 College of Horticulture and Landscape Architecture, Yangzhou University, Yangzhou 225009, China; zglu@yzu.edu.cn (Z.L.);; 2 College of Bioscience and Biotechnology, Yangzhou University, Yangzhou 225009, China

**Keywords:** long non-coding RNA, floral scent, terpenoid, phenylpropanoid, jasmine

## Abstract

Long non-coding RNAs (lncRNAs) have emerged as curial regulators of diverse biological processes in plants. Jasmine (*Jasminum sambac*) is a world-renowned ornamental plant for its attractive and exceptional flower fragrance. However, to date, no systematic screening of lncRNAs and their regulatory roles in the production of the floral fragrance of jasmine flowers has been reported. In this study, we identified a total of 31,079 novel lncRNAs based on an analysis of strand-specific RNA-Seq data from *J. sambac* flowers at different stages. The lncRNAs identified in jasmine flowers exhibited distinct characteristics compared with protein-coding genes (PCGs), including lower expression levels, shorter transcript lengths, and fewer exons. Certain jasmine lncRNAs possess detectable sequence conservation with other species. Expression analysis identified 2752 differentially expressed lncRNAs (DE_lncRNAs) and 8002 DE_PCGs in flowers at the full-blooming stage. DE_lncRNAs could potentially *cis*- and *trans*-regulate PCGs, among which DE_lincRNAs and their targets showed significant opposite expression patterns. The flowers at the full-blooming stage are specifically enriched with abundant phenylpropanoids and terpenoids potentially contributed by DE_lncRNA *cis*-regulated PCGs. Notably, we found that many *cis*-regulated DE_lncRNAs may be involved in terpenoid and phenylpropanoid/benzenoid biosynthesis pathways, which potentially contribute to the production of jasmine floral scents. Our study reports numerous jasmine lncRNAs and identifies floral-scent-biosynthesis-related lncRNAs, which highlights their potential functions in regulating the floral scent formation of jasmine and lays the foundations for future molecular breeding.

## 1. Introduction

The genome of eukaryotes comprises both protein-coding RNAs and non-coding RNAs (ncRNAs), whose interaction controls the expression pattern of genes [1]. Recent studies have revealed the essential roles of ncRNAs in regulating various biological processes in organisms such as cell development, regulation of epigenetics, transcription, and translation [2,3]. The ncRNAs involve rRNA (ribosomal RNA), tRNA (transfer RNA), snRNA (small nuclear RNA), snoRNA (small nucleolar RNA), miRNA (microRNA), lncRNA (long non-coding RNA), and other RNAs with known functions [3]. The lncRNA represents a large class from the ncRNA and contains more than 200 nucleotides but lacks the protein-coding ability [4]. Based on the genomic origin, the lncRNAs can be classified into three types: lincRNAs (long intergenic ncRNAs), incRNAs (intronic ncRNAs), and NATs (natural antisense transcripts) [5]. Additionally, the lncRNAs are present at relatively lower levels and exhibit little sequence conservation compared to mRNAs (messenger RNAs) [6]. LncRNAs inhibit the activity of miRNAs, thereby preventing the degradation of target mRNAs. LncRNAs also interact with DNA, RNA, and proteins, and are involved in the regulation of the functional activity of proteins and their modification, and chromatin remodeling [5,7].

Plant lncRNAs play important roles in different biological processes including gene silencing, seedling photomorphogenesis, flowering time, root organogenesis, reproduction, stress tolerance, and immunity [5]. For example, in *A. thaliana*, the accumulation of the lncRNA, *SABC1* (salicylic acid biogenesis controller 1), is reduced as a result of bacterial and viral infection. Consequently, the transcription of the *NAC3* gene activates salicylic acid biosynthesis, proving the involvement of the lncRNA *SABC1* in the plant immune response [8]. The *LncRNA.2-FL* plays a crucial role in the resistance of rice plants to salt stress and the formation of lateral roots by redistributing auxin concentration under salinity [9]. The two lncRNAs, termed *COOLAIR* (cool-assisted intronic non-coding RNA) and *COLDAIR* (cold-assisted intronic non-coding RNA), have been identified as key regulators of flowering time during vernalization in *Arabidopsis*, by negatively regulating a MADS-box transcription factor FLC (FLOWERING LOCUS C) [10]. The lncRNA *LDMAR* regulates the photoperiod-sensitive male sterility in rice plants [11]. In tomato plants, the lncRNA regulates the expression of neighboring genes influencing tomato fruit cracking [12]. In a recent study, 19 lncRNAs were predicted to participate in floral scent synthesis in *Rosa hybrida* [7]. However, the role of lncRNAs in the formation of floral scent is still largely unknown.

Flower scents possess various functions, and are mainly released to attract specific pollinators to facilitate sexual reproduction. The floral volatiles are also used in the production of several items such as food flavorings, perfumes, medicine, and many others [13]. As mentioned above, there is a lack of information of the regulatory roles of lncRNAs in floral scents. Therefore, to comprehend the role of lncRNAs in floral scents, the identification and functional validation of lncRNAs is crucial.

Jasmine (*Jasminum sambac*) is one of the most important ornamental plants, belonging to the family Oleaceae and cultivated in tropical and subtropical regions [14]. The jasmine plant is famous for its attractive, sweet-fragrant white flowers. Jasmine plants possess great economic value and are entered widely into the manufacturing of several products. The flowers and the extracted oils are used in several products such as perfumery, cosmetics, and food, etc. The jasmine tea is widely used in East Asia and is made from jasmine flowers. Moreover, the extracts are used in traditional medical treatments. Previous studies paid more attention to analyzing the chemical constituents of the jasmine floral scent, and around 100 chemical compounds have been identified. Among them, linalool, nerolidol, citronellol, β-farnesene, benzaldehyde, benzyl alcohol, and benzyl acetate are most frequent in the extracts of *J. sambac* flowers [15]. Generally, several jasmine-floral-scent-related genes have been discovered and functionally validated. For example, the genes *JsAOC1* and *JsAOS* in jasmonic acid biosynthesis and *JsTPS3* in β-ocimene biosynthesis were functionally validated [16]. However, the information of the regulatory mechanism is still largely unclear. In addition, none of these studies focused on the role of lncRNAs in the formation of jasmine floral scents. 

Here, we used the strand-specific RNA-seq (ssRNA-seq) in combination with a highly efficient computational pipeline to analyze and identify the lncRNAs in the *J. sambac* (cultivar ‘double petal’) flowers. Sequence conservation among species were explored. Two different developmental stages of jasmine flowers, full-blooming flower (FF) and flower bud (FB), were used. In addition, the expression patterns and potential regulatory roles of these lncRNAs in the emission of jasmine floral scent were examined. Our findings will be helpful in clarifying the regulatory roles of lncRNAs in the production of jasmine plant floral scents and facilitate the breeding of a fragrant plant with an improved aroma. 

## 2. Materials and Methods 

### 2.1. Plant Material and Growth Conditions

The plant material *J. sambac* ‘double petal’ cultivar was planted in the greenhouse of the campus of Yangzhou University, Yangzhou, China. According to the open state and aroma release of the flowers, we divided the flower development into two stages: (1) flower buds (FBs), of which the small petals are still closed and have little fragrance; and (2) full-blooming flowers (FFs), of which the petals are all unfolded and the fragrance is rich. All fresh flower samples from both stages were collected at 8:00 a.m. For strand-specific RNA-seq and qRT-PCR, 8–10 flower buds and 8–10 full-blooming flowers per plant were harvested from three healthy jasmine plants (three biological replicates). After that, the petals were isolated. All petal samples were immediately snap-frozen in liquid nitrogen and then stored in a freezer at −80 °C until use. 

### 2.2. RNA Isolation and Strand-Specific RNA Sequencing

The total RNA of each sample was extracted using a universal plant total RNA isolation kit (Vazyme Biotech Co., Ltd., Nanjing, China) according to the manufacturer’s instructions. RNA purity and concentration were examined using a NanoPhotometer^®^ spectrophotometer (NanoPhotometer; Implen, Calabasas, CA, USA), and the RNA integrity and quantity were finally measured using a Bioanalyzer 2100 system (Agilent, Carlsbad, CA, USA) with the RNA Nano 6000 Assay Kit.

Six strand-specific RNA libraries were prepared with ribosomal RNA (rRNA) depletion and standard lncRNA-seq methods. Briefly, the rRNA removal was conducted using the Ribo-Zero rRNA Removal Kit (Epicentre, Madison, WI, USA). Then, the rRNA-free sequencing libraries were generated using NEBNext^®^ Ultra™ II RNA Kits (NEB, Ipswich, MA, USA) following the manufacturer’s instructions. Next, 250–300 bp RNA fragments were generated using divalent cations at a high temperature in 5 × NEBNext First-Strand Synthesis Reaction Buffer. First-strand cDNA was synthesized using M-MuLV Reverse Transcriptase (RNaseH) with random hexamer primers, and, subsequently, second-strand cDNA was synthesized using DNA polymerase I, dNTPs, and RNaseH. Remaining overhang ends were converted via blunt using exonuclease or polymerase. After adenylation of the 3′ ends and ligation of the NEBNext adaptor, the library fragments were further purified with AMPure XP beads to preferentially select insert fragments of 250–300 bp in length. USER Enzyme (NEB, Ipswich, MA, USA) was added to digest the second strand with Uridine before PCR amplification. The concentration and quality of PCR products were determined by a Qubit^®^ fluorometer (Life Technologies, Carlsbad, CA, USA) and an Agilent Bioanalyzer 2100 system. The libraries were paired-end (PE150) sequenced on an Illumina Hiseq platform.

### 2.3. Identification and Classification of Novel lncRNAs in J. sambac

To obtain clean data, Illumina raw data were processed by removing the adapter sequences, poly-N-containing reads, and low-quality reads. The Q20, Q30, and GC content of the clean data were determined. All downstream analyses were based on high-quality, clean datasets obtained from six ssRNA-seq libraries. The clean data were mapped to our chromosome-level *J. sambac* genome [16] using Hisat2 (v2.0.5) [17]. The mapped reads were used for transcript assembly using StringTie (v1.3.3) with the parameter ‘--rf -e’, and then all transcripts with annotations were merged by Cuffmerge (v2.2.1). Cuffcompare software (v2.2.1) was used to compare the assembled transcripts with the jasmine genome annotation file, and the unknown transcripts that were annotated in the jasmine genome were retained for downstream analyses. The unknown transcripts were further filtered using the following steps: (1) removal of transcript length < 200 nt and without exon (exon number < 1); (2) removal of low-expressed transcripts with FPKM < 0.5; and (3) removal of the transcripts with protein-coding capability using CNCI [18], Pfam [19], and CPC2 [20] databases. The transcripts that did not have coding potential and overlapped within the three databases were considered the novel lncRNA dataset of *J. sambac*. Finally, the novel lncRNAs were annotated with the symbols ‘u’, ‘x’, ‘i’, and ‘o’, which represent intergenic, antisense, intronic, and sense lncRNAs. Novel lncRNAs were named according to the rules of the HGNC (The HUGO Gene Nomenclature Committee, Hinxton, UK). The characteristics (transcript length and exon number) of novel lncRNA were compared with those of known lncRNA and mRNA. The distribution and characteristics of these four types of lncRNAs on the 13 chromosomes of the jasmine genome were plotted using TBtools [21].

### 2.4. Homologous Analysis of Novel lncRNAs Compared with the Other Species

Sequences of lncRNAs from 23 plant species (*Arabidopsis thaliana*, *Brassica napus*, *Chenopodium quinoa*, *Corchorus capsularis*, *Chlamydomonas reinhardtii*, *Manihot esculenta*, cucumber, soybean, apple, *Medicago truncatula*, *Oryza sativa*, *Hordeum vulgare*, *Brachypodium distachyon*, *Ananas comosus*, *Amborella trichopoda*, *Selaginella moellendorffii*, *Physcomitrella patens*, *Chondrus crispus*, tomato, potato, cacao, grape, and maize) recorded in CANTATAdb 2.0 (http://rhesus.amu.edu.pl/CANTATA/index.html (accessed on 15 July 2023)) were retrieved and used to build blast databases. The jasmine lncRNA homologues in these species were identified using the blastn program with coverage ≥ 30% and an E-value < e^−10^, which were considered the conserved lncRNAs in *J. sambac*. Furthermore, the highly conservative lncRNAs were identified by the reciprocal blast between species with coverage ≥ 30% and an E-value < e^−10^.

### 2.5. Differential Expression Analysis of lncRNA and mRNA

The expression levels (FPKM) of both lncRNA transcripts and coding genes were calculated using StringTie software (v1.3.3). Differentially expressed lncRNAs (DE_lncRNAs) and protein-coding genes (DE_PCGs) were analyzed using the edgeR2 package. The *p*-values were adjusted using Benjamini and Hochberg’s approach for controlling the false discovery rate. LncRNAs or genes with |Log_2_ (fold change)| > 1 and an adjusted *p*-value (*q*-value) < 0.05 were identified as DE_lncRNAs or DE_PCGs.

### 2.6. Prediction and Annotation of Cis-Regulated and Trans-Regulated Target Genes of lncRNAs

Protein-coding genes (PCGs) located within a 100-kb window upstream or downstream of lncRNAs were identified as potential *cis*-regulated targets. PCGs can also exhibit a correlation in expression patterns with those of lncRNAs, and, thus, these co-expressed lncRNAs may regulate the PCGs in trans-regulation. Based on the Pearson’s correlation coefficients between lncRNAs and PCGs, the lncRNA-co-expressed PCGs were identified as potential *trans*-regulating targets. The DE_lncRNA *cis*-regulated and *trans*-regulated potential PCGs underwent functional enrichment analysis according to the GO and KEGG databases using the Goseq R package and KABAS software (V2.0). 

### 2.7. Metabolic Pathway Analysis

To investigate the biosynthesis of flower fragrances in *J. sambac*, we focused on the DE_lncRNA involved in the biosynthetic pathways of both terpenoids and phenylpropanoids. The targets of DE_lncRNAs were analyzed and visualized by Cytoscape (v.3.9.1). The heatmap was plotted using TBtools software (v2.030) [21]. 

### 2.8. Reverse-Transcription PCR (RT-PCR) and Quantitative Real-Time PCR (qRT–PCR)

The total RNA from the flower buds and full-blooming flowers used for ssRNA-seq was reverse-transcribed into cDNA using HiScript II Q RT SuperMix for qPCR with a gDNA wiper (Vazyme Biotech Co., Ltd., Nanjing, China). All designed gene-specific primers were checked using TBtools and then used in this study, as shown in Table S6. The specificity of the primer and the expected size of the PCR product were confirmed through the conduction of RT-PCR. To accurately quantify the changes in expression of the selected genes, qRT-PCR was performed on a CFX96 detection system (Bio-Rad, Hercules, CA, USA) following the procedures as described previously [22] with minor modifications. *JsActin2* (*JS2G6540*) was retrieved from previous *J. sambac* research [23,24] and used as an internal reference. All reactions were performed in three biological replicates, and the comparative threshold cycle (Ct) was determined. Relative expression levels of selected lncRNAs and genes were calculated using the 2^−ΔΔCt^ method [25]. 

## 3. Results

### 3.1. Floral Volatile Changes in J. sambac Flowers at Two Different Developmental Stages 

**Figure 1:**
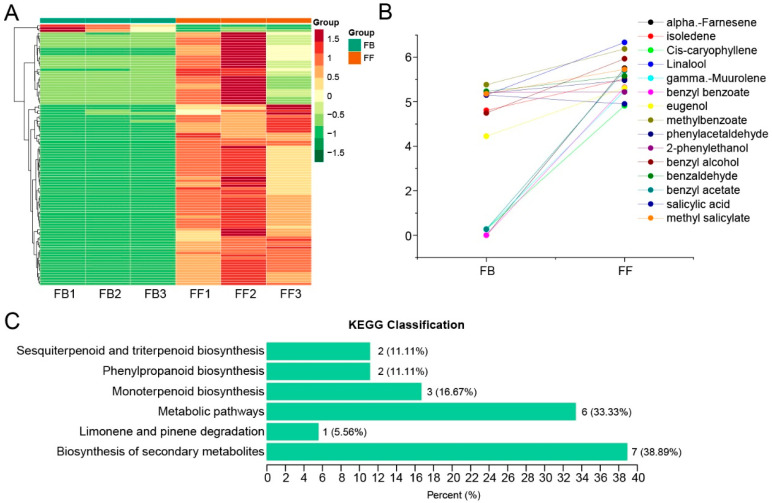
Identification and analysis of floral scent compounds in the flower buds (FBs) and full-blooming flowers (FFs). (**A**) Changes in metabolite contents detected by GC-MS. The dataset was retrieved from [16]. (**B**) Analysis of flower scent compounds that significantly changed in flowers from the FB to FF stage. (**C**) KEGG enrichment of all differential metabolites (18 metabolites) between FBs and FFs. The numbers on the bar indicate the number and proportion of enriched metabolites in each KEGG pathway.

The compositions and abundances of jasmine floral volatiles detected by GC-MS for two flowering stages (FBs and FFs) were retrieved from our previous study [16] and further analyzed. A total of 155 volatiles were detected (Table S1), and 108 of them significantly varied between FBs and FFs. Furthermore, 104 of them showed an upward trend from the FB to FF stages (Figure 1A). Importantly, some floral-scent-related volatiles, including terpenes (α-Farnesene, isoledene, cis-caryophyllene, Linalool, and γ-Muurolene) and phenylpropanoids (benzyl benzoate, eugenol, methylbenzoate, 2-phenylethanol, benzyl alcohol, benzaldehyde, benzyl acetate, salicylic acid, and methyl salicylate) increased significantly from the FB to FF stages (Figure 1B). Based on a Kyoto Encyclopedia of Genes and Genomes (KEGG) pathway analysis, the differentially abundant volatiles were classified in the biosynthesis of secondary metabolites, limonene and pinene degradation, sesquiterpenoid and triterpenoid biosynthesis, phenylpropanoid biosynthesis, monoterpenoid biosynthesis, and metabolic pathways (Figure 1C).

### 3.2. Identification and Characterization of Novel lncRNAs in J. sambac Flowers

**Figure 2:**
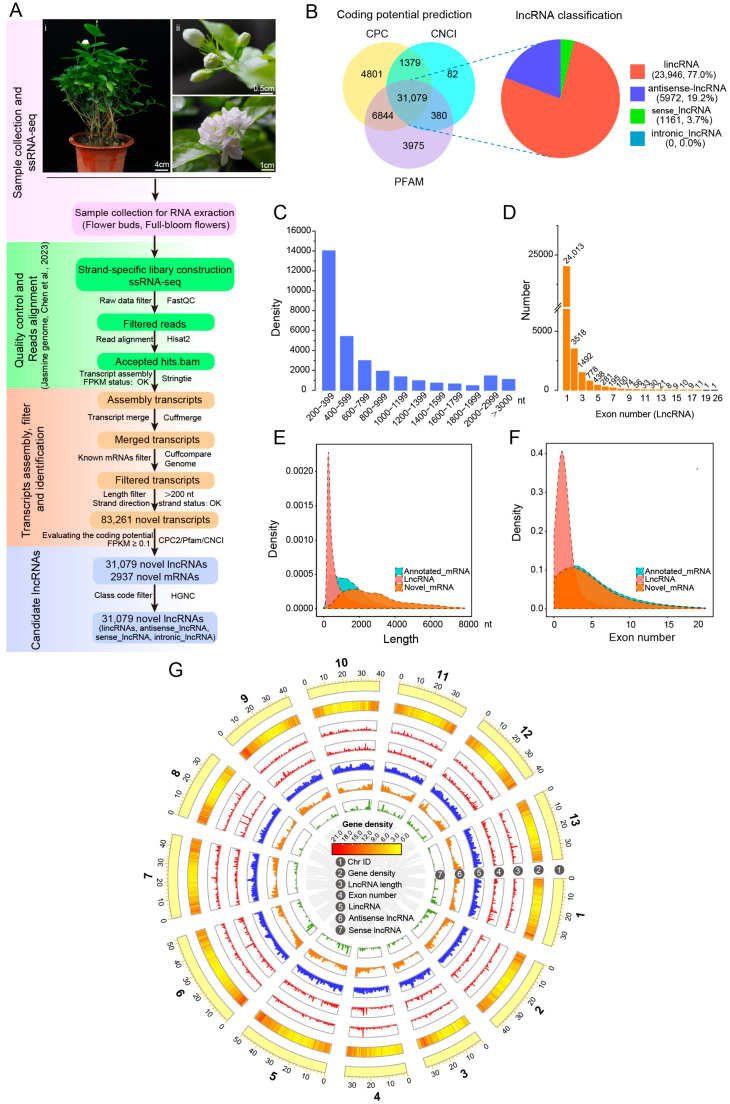
ssRNA-seq identification and characterization of lncRNAs in *J. sambac* flowers. (**A**) Schematic pipeline for the identification of lncRNAs in jasmine flowers. (**B**) Coding potential prediction and characterization of novel transcripts. The overlapped transcripts within the CNCI, Pfam, and CPC2 databases were considered the novel lncRNAs of jasmine. The candidate novel lncRNAs were classified into four types based on the relative position of lncRNAs to annotated transcripts in reference assembly. (**C**–**F**) Characterization and comparison of candidate lncRNAs and mRNAs. The length distribution (**C**,**E**) and exon number (**D**,**F**) were characterized and compared with annotated/novel mRNAs. (**G**) The circos plot shows the identified novel lncRNAs distributed in the *J. sambac* genome. The gray curve in the middle of the circos plot indicates the collinearity of protein-coding genes in the *J. sambac* genome.

To comprehend the landscape and expression pattern of lncRNAs in *J. sambac* flowers, six strand-specific RNA-seq (ssRNA-seq) libraries were constructed using the total RNA of flower buds and full-bloom flowers (Figure 2A), respectively, with three biological replicates for each. After trimming the adapters and low-quality reads, 18.79–24.69 GB of clean data for each library were obtained, with Q30 > 93.08% (Table S2). Then, the clean data were mapped to our chromosome-level *J. sambac* genome. The mapped reads were used for transcript assembly and annotation, and were categorized into four different classes, including intergenic, intronic, sense, and antisense transcripts. The unknown transcripts that were annotated in the jasmine genome were retained for downstream analyses. The unknown transcripts were further filtered with length < 200 without exons or with expression levels (FPKM) < 0.1, and 83,261 novel transcripts were retained. Then, three methods—CPC2, Pfam, and CNCI—were adopted to predict the non-coding transcripts, and 44,103, 42,278 and 32,920 putative non-coding transcripts were obtained, respectively (Figure 2B). Combining the results from the three databases, 31,079 confidence lncRNAs comprising 23,946 (77.0%) lincRNAs, 5972 (19.2%) antisense lncRNAs, 1161 (3.7%) sense lncRNAs, and 0 intronic lncRNAs expressed in *J. sambac* flowers were obtained (Figure 2A,B; Table S3). In addition, 2937 novel mRNAs were identified (Figure 2A).

To characterize the basic genomic features of the novel lncRNAs in *J. sambac* flowers, we analyzed the length distribution and exon number between lncRNAs and protein-coding transcripts. For lncRNAs, the lengths ranged from 200 to 19,884 nt, with an average length of 802 nt, and the majority (78.34%) were 201–1000 nt. By contrast, 54.79% of mRNAs were >1000 nt, and the average length of mRNAs was 2909 nt (Figure 2C,E). Gene structure analysis showed that most lncRNAs (24,013, 77.26%) contained one exon, followed by two exons (3518, 11.32%), whereas only 18.85% and 18.10% of mRNAs had one and two exons, respectively (Figure 2D,F). The average exon count of lncRNA transcripts (1.5 exons per transcript) was less than that of mRNAs (5.1 exons per transcript). These results suggest that, compared to mRNAs, lncRNAs in *J. sambac* flowers are relatively short and have fewer exons.

We characterized the basic genomic features of the obtained lncRNAs and compared these features with *J. sambac* protein-coding genes where appropriate. We found that the pericentromeric regions of most *J. sambac* chromosomes have a lower density of mRNAs, antisense lncRNAs, and sense lncRNAs than chromosome ‘arms’, while *J. sambac* lincRNAs are more evenly distributed across chromosomes (Figure 2G).

### 3.3. The Conservation of Novel lncRNAs in J. sambac

**Figure 3:**
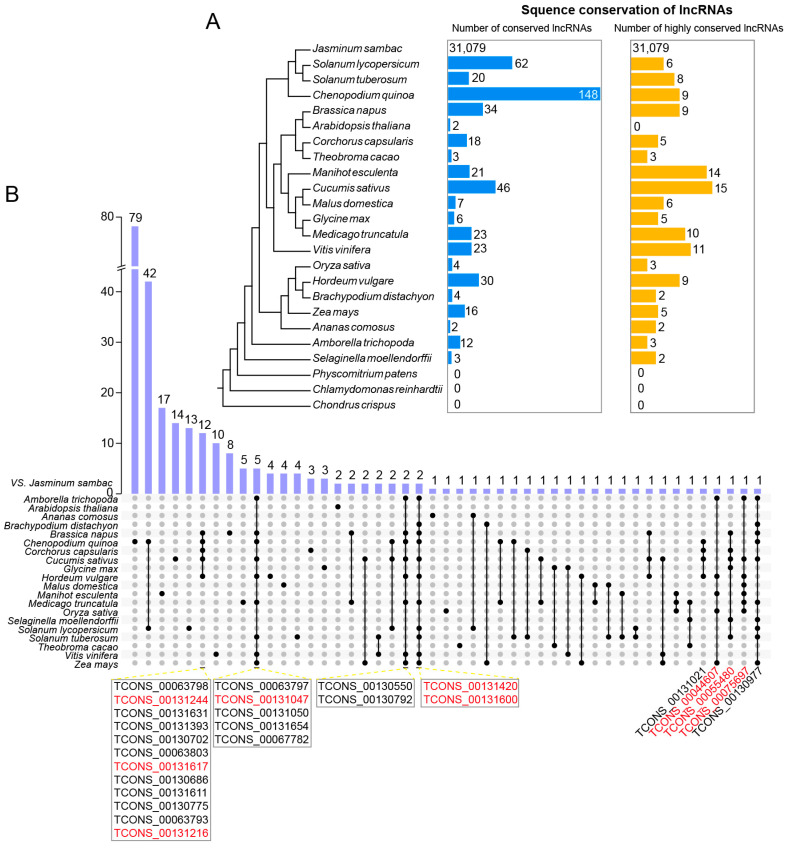
Sequence conservation analysis of novel lncRNAs in *J. sambac*. (**A**) Sequence conservation of lncRNAs in jasmine flowers and other 23 species. The blue column (left) indicates the number of homologues based on the sequence blast with an E-value < e^−10^ and coverage ≥ 0.3. The orange column (right) indicates the number of high-confident homologues based on the reciprocal blast with an E-value < e^−10^ and coverage ≥ 0.3. (**B**) Upset plot of all conserved lncRNAs between *J. sambac* and the other 23 species. The conserved jasmine lncRNAs shared with more than five species were listed, and the lncRNA highlighted by red color was the high-confident conserved lncRNAs between species.

To gain more insights into the evolution and conservation of lncRNAs in *J. sambac* flowers, we conducted a BLAST search to detect homology using lncRNAs from 23 species, including eudicots, monocots, basal angiosperms, ferns, mosses, and green algae (Figure 3A). Based on the phylogenetic tree of the 24 species, the most conserved lncRNAs (148) were found in *C. quinoa*, followed by *Solanum lycopersicum* (62). Homologous lncRNAs were found in some eudicots, but they were absent in basal angiosperm (*C. crispus*), moss (*P. patens*), and green algae (*Chlamydomonasre inhardtii*). To obtain highly conserved lncRNAs, we performed the reciprocal blast with an E-value < e^−10^ and coverage ≥ 0.3. The highest number was found in *Cucumis sativus* (15), followed by *Manihotes culenta* (14). None of the highly conserved lncRNAs were found in *C. crispus*, *P. patens*, *C. inhardtii,* and *A. thaliana* (Figure 3A). Some highly conserved lncRNAs were found to be common in several species, such as TCONS_0013131244 and TCONS_00131617 in *J. sambac*, *B. napus*, *C. quinoa*, *C. capsularis,* and *C. sativus*; and TCONS_00131420 and TCONS_00131600 in *J. sambac*, *B. distachyon*, *B. napus*, *C. quinoa*, *C. sativus*, *H. vulgare*, *M. truncatula*, *S. lycopersicum*, *S. tuberosum*, *V. vinifera,* and *Z. mays* (Figure 3B). 

### 3.4. Expression of lncRNAs and mRNAs in Different Developmental Stages of J. sambac Flowers

**Figure 4:**
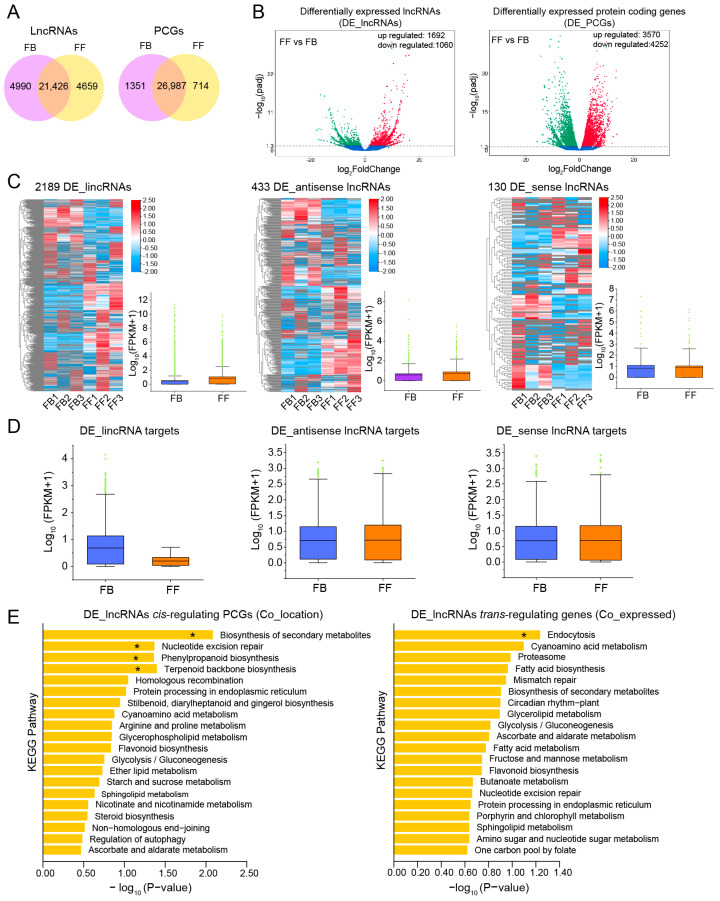
Identification of differentially expressed lncRNAs (DE_lncRNAs) and their potential protein-coding genes (PCGs). (**A**) The commonly and specifically expressed lncRNAs/PCGs between FBs and FFs. (**B**) Volcano plot showing differentially expressed lncRNAs (DE_lncRNAs, upregulated and downregulated) and differentially expressed PCGs (DE_PCGs, upregulated and downregulated). (**C**) Heatmap and boxplot showing the expression changes of DE_ lincRNAs, DE_antisense lncRNAs, and DE_sense lncRNAs between FBs and FFs. (**D**) Expression change of the DE_lncRNA (lincRNA, antisense lncRNAs, and sense lncRNA) potentially targeted PCGs. (**E**) KEGG enrichments of DE_lncRNA *cis*-regulated PCGs (Co_location) and *trans*-regulated (Co_expression) PCGs. The significantly enriched pathway was marked by an asterisk (*).

We then systematically estimated the expression level of all transcripts identified, including lncRNAs and protein-coding genes (PCGs), using FPKM. In both FBs and FFs, there were 21,426 and 26,987 commonly expressed lncRNAs and PCGs, respectively (Figure 4A). Then we identified significant differentially expressed lncRNAs (DE_lncRNAs) and DE_PCGs between FBs and FFs. The results indicated that 1692 lncRNAs presented a significantly upregulated pattern, while 1060 lncRNAs were significantly downregulated in FFs compared to FBs (Figure 4B). In addition, compared with FB, 8002 DE_PCGs were differentially expressed in FFs, including 3750 up- and 4252 downregulated PCGs (Figure 4B). To further investigate the potential function of these DE_PCGs in *J. sambac* flowers, Gene Ontology (GO) and Kyoto Encyclopedia of Genes and Genomes (KEGG) enrichment analyses were performed. The significantly enriched GO terms and KEGG pathways were analyzed (Figure S1). Among the enriched GO terms, “biological process” and “catalytic activity” were significantly enriched in the FFs vs. FBs (Figure S1A). Among the enriched KEGG pathways, “terpenoid backbone biosynthesis”, “sesquiterpenoid and triterpenoid biosynthesis”, “phenylpropanoid biosynthesis”, “diterpenoid biosynthesis” and “biosynthesis of secondary metabolites” were significantly enriched (Figure S1B). 

We further analyzed the expression patterns of different types of lncRNAs. Overall, we recorded 2189 DE_lincRNAs, 433 DE_antisense lncRNAs, and 130 DE_sense lncRNAs (Figure 4C). The average expression levels of DE_lincRNAs, DE_antisense lncRNAs, and DE_sense lncRNAs were all higher in FFs than in FBs (Figure 4C). LncRNAs have been found to function in regulating the expression of proximal and distal PCGs through *cis*- and *trans*-acting mechanisms [26,27]. In total, 13,773, 5828, and 1692 genes were predicted to be target genes of DE_lincRNAs, DE_antisense lncRNAs, and DE_sense lncRNAs, respectively. The overall expression levels of these target genes were estimated for FBs and FFs. As shown in Figure 4D, the mean expression levels of DE_lincRNA targets were lower in FFs than in FBs, while the targets of DE_antisense lncRNAs and DE_sense lncRNAs showed the opposite expression patterns. 

One of the ways lncRNAs function is by affecting neighbor gene expression levels, and the PCGs located within a 100 kb window upstream or downstream of lncRNAs were identified as potential cis-regulated targets. Following these criteria, 339 targets were obtained for 26 DE_lncRNAs, with an average of 13 candidate targets per DE_lncRNA (Table S5). Meanwhile, trans-targets were also predicted by screening the lncRNA–mRNA pairs, depending on the Pearson correlation coefficients. In this way, 16,948 mRNAs were found to potentially interact with 2189 DE_lncRNAs. To understand the possible functions of the DE_lncRNAs in flowers, KEGG annotation and enrichment of the putative lncRNA *cis*- and *trans*-targets were performed. As shown in Figure 4E, the “biosynthesis of secondary metabolites”, “nucleotide excision repair”, “phenylpropanoid biosynthesis”, and “terpenoid backbone biosynthesis” pathways were significantly enriched in *cis*-targets, while only the pathway of “endocytosis” was significantly enriched in *trans*-regulated genes. Given that terpenoid- and phenylpropanoid-related secondary metabolites are the important components of floral scent biosynthesis, we concentrated on the role of lncRNA *cis*-regulated PCGs in floral scent biosynthesis. 

### 3.5. Identification and Association of lncRNAs with Floral Scent Biosynthesis Pathways

**Figure 5:**
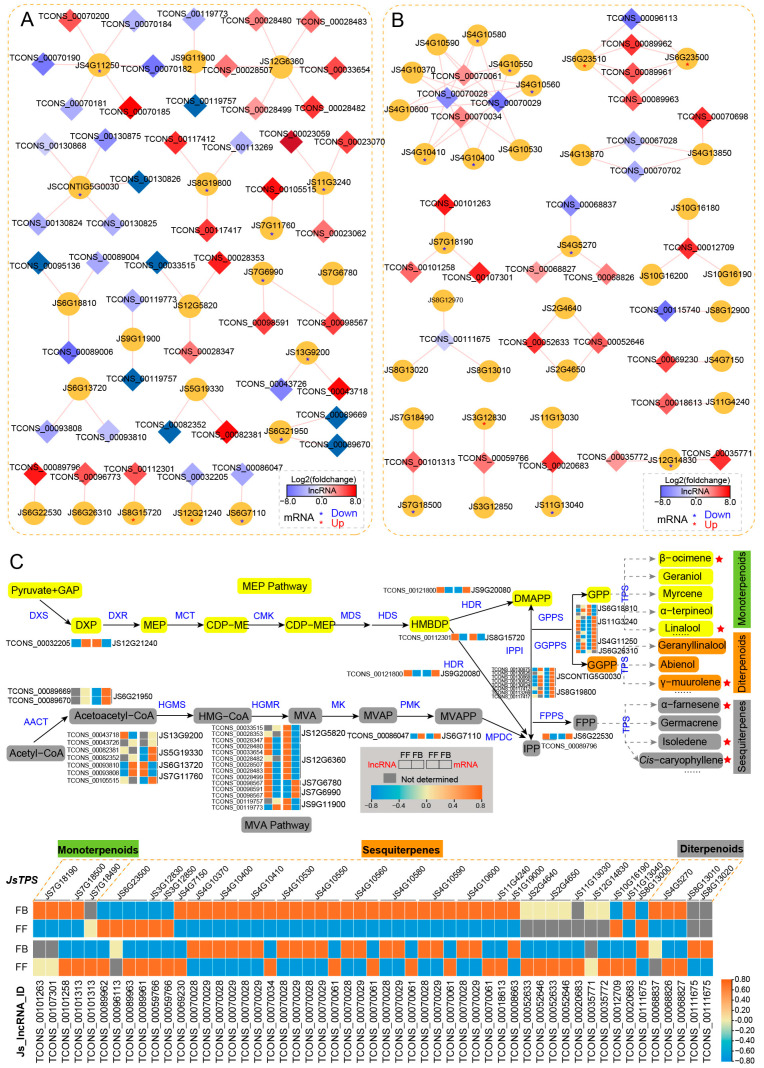
*Cis*-regulated DE_lncRNAs and their PCGs involved in the terpene biosynthesis pathways of *J. sambac*. (**A**,**B**) DE_lncRNAs *cis*-regulated MEP and MEV pathway-related genes (**A**) and *JsTPS* genes (**B**) involved in terpene biosynthesis. The DE_lncRNA–PCG network was constructed by Cytoscape v3.9.1. The diamond indicates DE_lncRNA, and the cycle indicates PCG. The diamond with red color represents the significantly upregulated lncRNA in FFs, and the blue color represents the significantly downregulated lncRNA. The red and blue asterisks (*) within the cycle indicate that the mRNA is significantly upregulated and downregulated in FFs, respectively. (**C**) LncRNA *cis*-regulating mechanism portrait of terpene biosynthesis pathways illustrating the terpene compound formation of jasmine flower scents. Both MEP and MVA pathways are shown, and the expression changes of DE_lncRNA and their target PCGs are displayed by heatmap. The volatile terpenes (detected by GC-MS) significantly upregulated in FFs are marked in red stars.

**Figure 6:**
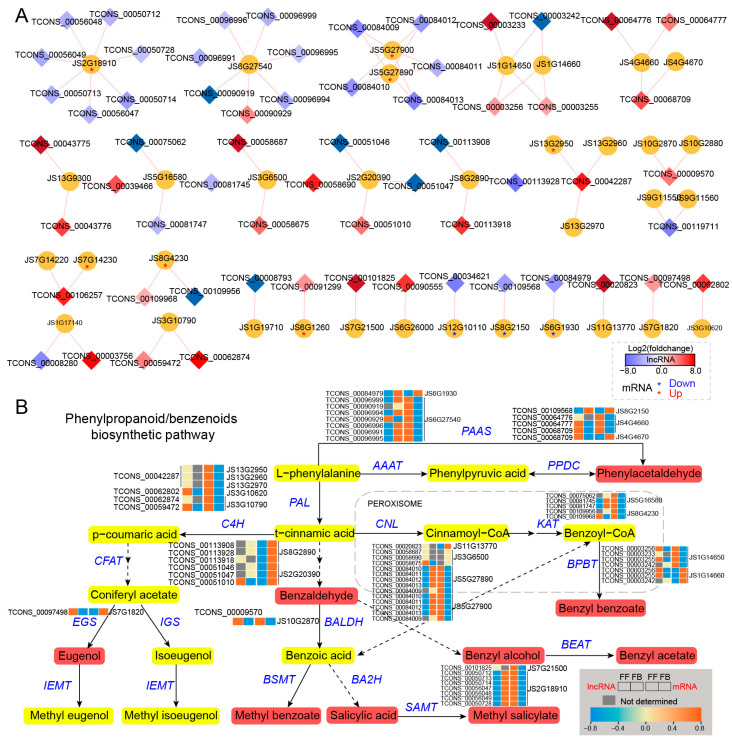
*Cis*-regulated DE_lncRNAs and their PCGs involved in phenylpropanoid/benzenoid pathways of *J. sambac*. (**A**) DE_lncRNAs *cis*-regulated target genes involved in phenylpropanoid/benzenoid biosynthesis. The DE_lncRNA-PCG network was constructed by Cytoscape v3.9.1. The diamond indicates DE_lncRNA, and the cycle indicates PCG. The diamond with a red color represents the significantly upregulated lncRNA in FFs, and the blue color represents the significantly downregulated lncRNA. The red and blue asterisks (*) within the cycle indicate the significantly upregulated and downregulated mRNA in FFs, respectively. (**B**) LncRNA *cis*-regulating mechanism portrait of the phenylpropanoid/benzenoid biosynthesis pathway illustrating the phenylpropanoid/benzenoid compound formation of jasmine flower scents. Expression changes of DE_lncRNA and their target PCGs are displayed by heatmap. The content of phenylpropanoid/benzenoid components (detected by GC-MS) significantly increased in FFs, which is marked in red.

Flower fragrances are volatile organic compounds (VOCs), and the VOCs of *J. sambac* floral scents mainly belong to the terpenoid and phenylpropanoid/benzenoid classes. To reveal the potential regulatory roles of lncRNAs in floral scent, we focused on analyzing the DE_lncRNAs and their *cis*-targets involved in terpenoid (MEP and MVA pathways) and phenylpropanoid/benzenoid biosynthesis pathways (Figure 5 and Figure 6). In the terpenoid biosynthesis pathway, nine genes (*DXS*, *HDR*, *IPPI*, *GPPS*, and *GGPPS*) were predicted to be putative targets of 24 lncRNAs in the MEP pathway (Figure 5A). For instance, four putative targets were annotated to be *GPPS*, among which *JS11G3240*, *JS4G11250,* and *JS6G26310* were shown to be upregulated in FBs. In contract, most of the corresponding lncRNAs were downregulated. In the MVA pathway, 12 putative targets were predicted for 25 DE_lncRNAs. Many of these target genes are more highly expressed in FBs than in FFs, such as three genes encoding HGMS (*JS13G9200*, *JS5G19330,* and *JS7G11760*), one *AACT* gene (*JS6G21950*), two *HGMR* genes (*JS7G6780* and *JS7G6990*) and one *PMK* gene (*JS6G7110*). Among the DE_lncRNAs in the MVA pathway, 15 were upregulated and 10 were downregulated in FFs. 

The terpene synthase (TPS) family is a vital enzyme gene family for terpene biosynthesis, which is crucial in the production of floral VOCs. We identified 34 *TPS* genes as potential targets of 65 DE_lncRNAs (Figure 5B). Among them, 13 *TPS* genes targeted by 18 lncRNAs were differentially expressed between FFs and FBs. More importantly, four *TPS* (*JS6G23500*, *JS6G23510*, *JS3G12830*, and *JS3G12850*) genes (targeted by) encoding synthetases that potentially regulate the synthesis of sesquiterpenes (α-farnesene and germacrene), as well as the corresponding six DE_lncRNAs, were almost all expressed at significantly higher levels in FFs than in FBs, which may promote the synthesis of sesquiterpene compounds contributing to floral fragrance, as indicated by the abundance of α-farnesene, isoledene, and cis-caryophyllene in FFs. By contrast, nine *TPS* genes (targeted by 12 DE_lncRNAs) involved in the biosynthesis of other sesquiterpene (e.g., isoledene), monoterpenes (β-ocimene and linalool), and diterpenoids (γ-muurolene), as well as their corresponding 12 DE_lncRNAs, were mostly expressed at lower levels in FFs than in FBs. For example, *JS7G18190*, *JS7G18500,* and *JS7G18490* were responsible for monoterpene synthesis, and they were target genes of TCONS_00101263, TCONS_00107301, TCONS_00101258, TCONS_00101313, and TCONS_00101313, of which were less expressed in FFs than in FBs. Although the abundant terpenoid compounds were accumulated in FFs, the relative higher expression of *TPSs* in FBs may promote the biosynthesis of terpenoid compounds early but accumulated or released in FFs. 

In the phenylpropanoid/benzenoid biosynthesis pathway, a total of 35 genes were predicted to be targets of 61 lncRNAs, and their interaction networks are shown in Figure 6A. Among them, most DE_lncRNAs were upregulated, whereas their putative *cis*-targets were downregulated in FBs compared with FFs, for example, TCONS_00096999, TCONS_00090919, TCONS_00096994, TCONS_00096996, TCONS_00096991, and TCONS_00096995. The predicted target genes for them were *JS6G27540*, which encodes aromatic amino acid aminotransferase (AAAT), whose expression level was lower in FBs than in FFs. Likewise, of the upregulated lncRNAs (FFs vs. FBs), TCONS_00084010, TCONS_00084011, TCONS_00084012, TCONS_00084013, TCONS_00084009, TCONS_00084010, TCONS_00084011, TCONS_00084012, TCONS_00084013, and TCONS_0008400 were targeted at two downregulated genes encoding cinnamate-coenzyme A ligase (CNL, *JS5G27890* and *JS5G27900*). In addition, two genes encoding 3-ketoacyl-CoA-thiolase (KAT) had higher expression levels in FFs than in FBs, and they were predicted to be the targets of five lncRNAs, four of which showed a lower expression in FFs. At the downstream of the phenylpropanoid/benzenoid pathway, seven upregulated lncRNAs (TCONS_00050712, TCONS_00050713, TCONS_00050714, TCONS_00056047, TCONS_00056048, TCONS_00056049, and TCONS_00050728) were targeted at genes encoding salicylic acid carboxyl methyltransferase (SAMT, *JS2G18910*). The expression level of the *SAMT* (*JS2G18910*) gene was significantly higher in FFs (Figure 6B). 

### 3.6. qRT-PCR Validation of lncRNAs Involved in Floral Scent Biosynthesis

**Figure 7:**
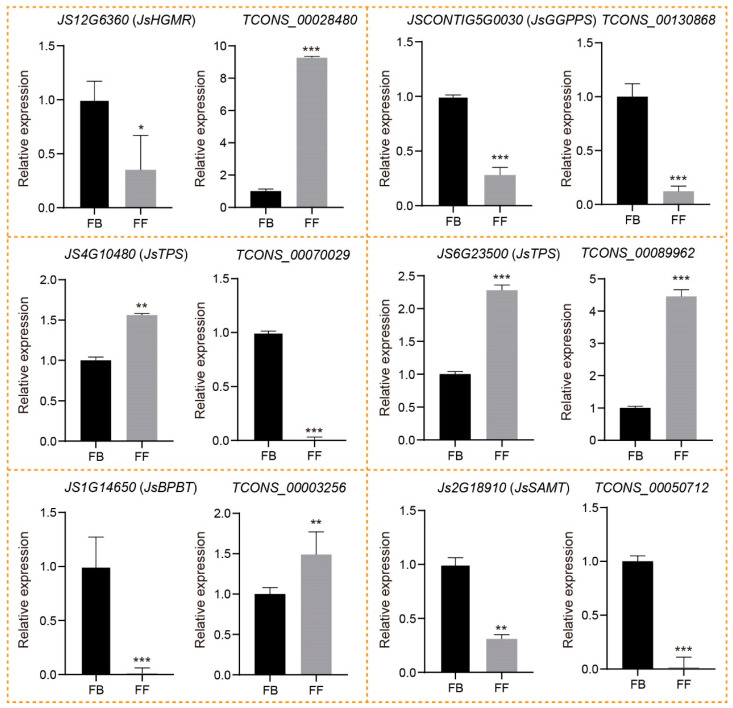
Expression validation of lncRNAs and their potential *cis*-regulated targets with qRT-PCR in two flowering stages (FBs and FFs). The data are presented as means, with error bars indicating SE. The asterisk indicates a significant difference according to Student *t*-test. * *p* < 0.05; ** *p* < 0.01; *** *p* < 0.001.

To verify and identify the candidate lncRNAs;’*cis*-regulated potential PCGs involved in floral scent biosynthesis, we first conducted RT-PCR to examine the specificity of the primers for six DE_lncRNAs and the corresponding DE_PCGs selected from both the terpenoid and phenylpropanoid/benzenoid pathways. All candidate lncRNAs and PCGs were successfully amplified and showed clear bands on gel electrophoresis (Figure S2), confirming that our designed primers are specific to these candidate lncRNAs and mRNAs. To further identify their expression changes, we performed qRT-PCR of the six DE_lncRNAs and corresponding DE_PCGs during the release of *J. sambac* floral scent. The qRT-PCR analysis indicated that all six lncRNAs displayed similar trends of expression, as indicated by their corresponding FPKM values (Figure 7; Table S3). In the terpenoid biosynthesis pathway, the expression of TCONS_00028480 was significantly upregulated in FFs compared to FBs, whereas its *cis*-regulated PCGs encoding *JsHGMR* (*JS12G6360*) was decreased. Similarly, TCONS_00070029 and its target *JsTPS* (*JS4G10480*) also exhibit the opposite expression trends during the FB to FF stages. In contrast, both TCONS_00130868 and *JsGGPPS* (*JSCONTIG5G0030*) showed the same expression patterns between FBs and FFs. Notably, *JsTPS* (*JS6G23500*) encoding α-farnesene synthetase was predicted as the target of TCONS_00089962, both of which displayed a significantly increased expression in FFs compared to FBs. Consistent with the abundance of linalool in FFs, the result suggested that TCONS_00089962 might have a positive role in the α-farnesene biosynthesis of *J. sambac*. In the phenylpropanoid/benzenoid biosynthesis pathway, the *JsBPBT* (*JS1G14650*) encoding putative benzoyl-CoA: benzylalcohol/2-phenylethanol benzoyltransferase was significantly downregulated in FFs, whereas its corresponding lncRNA TCONS_00003256 was expressed higher in FFs than FBs, in accordance with the greater accumulation of benzylbenzoate in FFs (Figure 1A and Figure 7). Moreover, TCONS_00050712 and its potential target, *JsSAMT* (*JS2G18910*), were both significantly downregulated in FFs compared to FBs. 

## 4. Discussion

Floral fragrance, one of the most significant ornamental characteristics, is essential for attracting pollinators and ensuring the reproductive success of flowering plants [28,29]. Different flowering plants possess distinct flower fragrances, which vary depending on the composition, quantity, and emission of floral volatile compounds (VOCs) [28,30]. For example, in Bulgarian rose, the most abundant components are phenethyl alcohol, citronellol, and heneicosane, whereas linalool (monoterpene), (3E, 6E)-a-farnesene (sesquiterpene), and benzyl acetate are major compounds of the floral scent in *Jasminum* species [31]. Indeed, our previous studies have revealed that the terpene (monoterpene and sesquiterpene) and phenylpropanoid/benzenoid (benzyl alcohol, eugenol, and benzyl benzoate) compounds are major components of VOCs in *J. sambac* flowers [16]. The floral VOC production markedly changes during flowering development [29]. In general, the flowers did not emit fragrance until they arrived at a state where the petals were opened and stamens were exposed thoroughly; most fragrance compounds are emitted and peaked in such a full-blooming stage. In the present study, we retrieved the previous metabolic data of GC-MS [16] and further performed a comprehensive comparison of metabolic components between the FB and FF stages. As expected, more than 100 floral volatiles significantly accumulated in the FF stage compared to the FB stage, further suggesting the emissions of VOCs were abundant in the FF stage. Importantly, most of the floral VOCs significantly enriched in the biosynthesis of terpenoid and phenylpropanoid exhibited a marked increase in the FF stage, indicating that the biosynthesis of terpenoid and phenylpropanoid plays a crucial role in the production of floral scents in *J. sambac*. In addition, the flowers at the FB and FF stages can be used as a comparative frame to investigate the regulatory mechanism of floral scent formation in jasmine flowers. 

With the advance of high-throughput sequencing technology, many novel non-coding RNA transcripts have been discovered and characterized from different species [7,32,33,34,35,36,37,38,39,40]. However, limited studies are available on the identification and characterization of lncRNAs in floral scent synthesis. Based on a combination of the ssRNA-seq and criteria pipelines widely used in other plants [7,33,41], we systematically identified a total of 31,079 novel lncRNAs in both flower buds and full-blooming flowers of *J. sambac*, a famous fragrant plant with sweet-scented flowers [16]. The number of potential lncRNAs (31,079) in *J. sambac* flowers was far greater than that of lncRNAs in Arabidopsis, rice, or rose [7,32,42], but less than that of lncRNAs (33,400) in black tea [39]. LncRNAs have been found to be potentially involved in the aroma formation of plants such as tea leaves and rose flowers [7,39]. The high number of lncRNAs identified in *J. sambac* flowers can be attributed to the fact that the synthesis of aroma compounds is more active and the productions are significantly accumulated from the bud to the full-blooming stage. In addition, although a large amount of publicly available transcriptome data and corresponding filtering criteria can be employed to identify novel lncRNAs, it is difficult to distinguish between sense and antisense strands of lncRNAs based only on common RNA-seq library construction and sequencing. This leads to quite a lack of lncRNAs information. However, the application of ssRNA-seq could facilitate the identification of the lncRNAs, providing valuable strand orientation information in various plant species [43,44]. Accordingly, in this study, 5972 antisense lncRNAs and 1161 sense lncRNAs were obtained in *J. sambac* flowers. Compared with mRNAs, we observed that most lncRNAs had 1–2 exons and exhibited lower expression levels, which is consistent with the characteristics of lncRNAs identified in other plants [32,39,40,42,45]. In summary, to our knowledge, this is the first work to globally identify lncRNAs that are involved in the flower-opening process and aroma formation in *J. sambac*. Our analysis generated a comprehensive list of potential lncRNAs for *Jasminum* species, which will greatly aid functional genomics research.

LncRNAs generally exhibited high divergence at the nucleotide level but relative higher conservation by position across species. In animals and plants, research has indicated that only 2–5.5% of lncRNAs exhibit evolutionary conservation in terms of primary sequences [5]. Here, we observed that only 484 jasmine lncRNAs (1.6%) were homologous with those of the other species, indicating a rapid evolutionary speed after jasmine speciation. Nevertheless, 127 lncRNAs were found to be highly conserved with other species based on reciprocal blast, among which six lncRNAs have the best homologous hits with those in at least four or more species, implying that these conserved lncRNAs may have relatively conserved functions among species (Table S7). As little lncRNA information is available within the Oleaceae family or *Jasminum* species, the exploration of the sequence conservation of lncRNAs among different species in the same family or genus needs to be further investigated in the future. 

A few studies indicated that lncRNAs are involved in the formation of aroma emitted by flowers, leaves, or other organs [7,39]. In rose, 103 differentially expressed lncRNAs were identified as potentially involved in floral scent production. In this study, we found that the expression levels of lncRNAs significantly changed (DE_lncRNAs) during the production of flower scent (from the flower bud to full-blooming stage), suggesting their important roles in regulating *J. sambac* flower scent production. In addition, lncRNAs can regulate the expression of PCGs via either *cis*- or *trans*-action [46]. Cis-acting lncRNAs were reported to regulate the expression of genes that are located in the vicinity of their transcription sites [47]. Moreover, lncRNAs can act as long-distance regulatory elements for controlling gene expression. For instance, the maize non-coding RNA *Vgt1*, located 70 kb upstream of the *ZmRap2* gene, influenced the expression of the *ZmRap2* gene. In the present study, we performed a genome-wide investigation of lncRNAs and PCGs that are adjacent to lncRNAs (<10 kb). Notably, the expressions of many lincRNAs were upregulated in the FF stage, while their neighboring PCGs (potential *cis*-regulated targets) were downregulated, indicating a negative correlation between the expression of lincRNA and nearby PCGs that both potentially participate in the formation of flower scent in *J. sambac*. Trans-acting lncRNAs could also regulate gene expression in a locus-specific manner [26,48]. We found that 433 differentially expressed antisense (DE_antisense) lncRNAs and their potential target PCGs exhibited significant changes in expression between the FB and FF. Compared with DE_lncRNA *trans*-regulating PCGs, a great number of *cis*-regulated PCGs involved in the biosynthesis of secondary metabolites, including phenylpropanoids, and terpenoid were found to be specifically enriched during the formation of jasmine flower scents. Given that the aroma compounds dramatically increased from the FB to FF stage (Figure 1, [16]), our present results suggest that lncRNAs implement divergent regulatory mechanisms to participate in the biosynthesis of floral scents in *J. sambac*.

Terpenoids are the most diverse group of volatile compounds in plants [49,50]. Based on the isoprenoid units, terpenoids can be classified as monoterpenes, sesquiterpenes, diterpenes, and others. Studies have revealed that the mevalonic acid (MVA) and 2-c-methylerythritol 4-phosphate (MEP) pathways are responsible for the synthesis of monoterpenes, sesquiterpenes, and diterpenes consisting of terpene aromatic compounds in plant flowers and fruits [13]. In fragrant plants, floral volatiles responsible for terpenoid synthases (TPSs) have been well-identified and characterized [13,51,52]. For example, in *Lilium* “Siberia” species, LoTPS2, LoTPS4, and LoTPS5 can catalyze the formation of (E, E)-α-farnesene, D-limonene, β-myrcene, and squalene, respectively [53,54]. In *Antirrhinum majus*, terpene synthase AmNES/LIS-1 is responsible for the biosynthesis of linalool, and AmNES/LIS-2 accounts for nerolidol formation in snapdragon flowers [51]. Recently, the role of *JsTPS3* was demonstrated to be involved in the β-ocimene biosynthesis of *J. sambac*. Although lncRNAs have various roles in plant physiological processes, their functions in aromatic compound synthesis have yet to be explored. In this study, we comprehensively analyzed the potential relationship between DE_lncRNAs and their co-location *TPS* genes involved in the terpenoid synthesis pathways (MEP and MVA) of *J. sambac* flowers. Many *TPSs* could be targeted by DE_lncRNAs, indicating that these lncRNAs may participate in the biosynthetic pathways of various aromatic terpenoids through *cis*-regulatory effects in *J. sambac*. Further expression analysis and qRT-PCR validation confirmed the expression correlation (negative or positive) between lncRNAs and their co-located terpenoid-synthesis-related genes (*JsTPS*s, *JsGGPPS*, and *JsHGMR*) during the FB to FF stage. Notably, TCONS_00089962 and its potential *cis*-regulated *JsTPS* gene, which encodes α-farnesene synthase, exhibited markedly increased expression in the FFs, suggesting that TCONS_00089962 may play an important role in promoting α-farnesene accumulation in the full-blooming flowers of *J. sambac* [16]. In addition, phenylpropanoids and benzenoids are the second-largest group of plant VOCs [55]. Our previous study has revealed a number of genes and metabolites in the biosynthetic pathway of phenylpropanoid/benzenoid in *J. sambac* [16]. Accordingly, in this study, many phenylpropanoid/benzenoid-biosynthesis-related genes were predicted to be potential targets of DE_lncRNAs, and both exhibited opposite expression trends from the FB to FF stages, indicating that these lncRNAs may also be involved in the phenylpropanoid/benzenoid biosynthesis of *J. sambac*. Collectively, our study provides valuable information on the lncRNAs involved in the biosynthesis of floral scents, whose function needs to be further validated by functional experiments in the future.

## 5. Conclusions

As the first systematically identified lncRNAs in *J. sambac* flowers, a total of 31,079 confidence lncRNAs comprising 23,946 lincRNAs, 5972 antisense lncRNAs, and 1161 sense lncRNAs were comprehensively identified and characterized. Although numerous jasmine lncRNAs showed low sequence similarities with those from other species, certain jasmine lncRNAs were highly conserved, implying their potential conserved roles in biological functions. Moreover, 2752 DE_lncRNAs were found in *J. sambac* flowers from the FB to FF stages, which suggests their potential roles in the release of jasmine flower scents. More importantly, an analysis of lncRNAs and their target genes revealed that *cis*-regulated lncRNAs were significantly involved in terpenoid and phenylpropanoid/benzenoid biosynthesis pathways. Furthermore, we constructed the regulatory network of flower-scent-related lncRNAs and their targets and compiled them into the terpenoid and phenylpropanoid/benzenoid biosynthesis pathway dynamics portrait. The *cis*-regulated lncRNAs and their *JsTPS* targets contributing to the formation of flower scents were highlighted. Overall, our investigation provides valuable gene resources for further improving floral scent in jasmine and offers new insights into the lncRNA regulations of floral scent synthesis in fragrant plants.

## Data Availability

The raw datasets of ssRNA-seq have been deposited in the Genome Sequence Archive (GSA) with the accession number CRA013543 (run accession number: CRR948016, CRR948017, CRR948018, CRR948019, CRR948020, and CRR948021 included) in the National Genomics Data Center.

## Supplementary Materials

The following supporting information can be downloaded at: https://www.mdpi.com/article/10.3390/biom14010045/s1, Figure S1: GO and KEGG enrichments of differentially expressed protein-coding genes (DE_PCGs) between flower buds (FBs) and full-blooming flowers (FFs) in J. sambac. Figure S2: Gel image of examination of primer specificity and expression of selected lncRNAs and mRNAs in the flower bud (FB) and full-blooming flower (FF) in J. sambac. Table S1: Volatile metabolites (GC-MS) in flower buds and full-blooming flowers in J. sambac. Table S2: Summary of ssRNA-seq data from flower buds (FBs) and full-blooming flowers (FFs). Table S3: Identification of novel lncRNAs in J. sambac flowers. Table S4: Sequence-conserved lncRNAs in J. sambac. Table S5: Differentially expressed lncRNAs (DE_lncRNAs) between flower buds (FBs) and full-blooming flowers (FFs). Table S6: List of the primers used in this study. Table S7: Cis-targets of conserved lncRNAs (Co_location mRNAs) and their function annotation.
