# Supplementary figures and images for: Genome-Wide Identification and Characterization of Long Non-Coding RNAs Associated with Floral Scent Formation in Jasmine (Jasminum sambac)
Source: Biomolecules. 2023 Dec 28;14(1):45. doi: 10.3390/biom14010045 (PMC10812929; doi:10.3390/biom14010045)

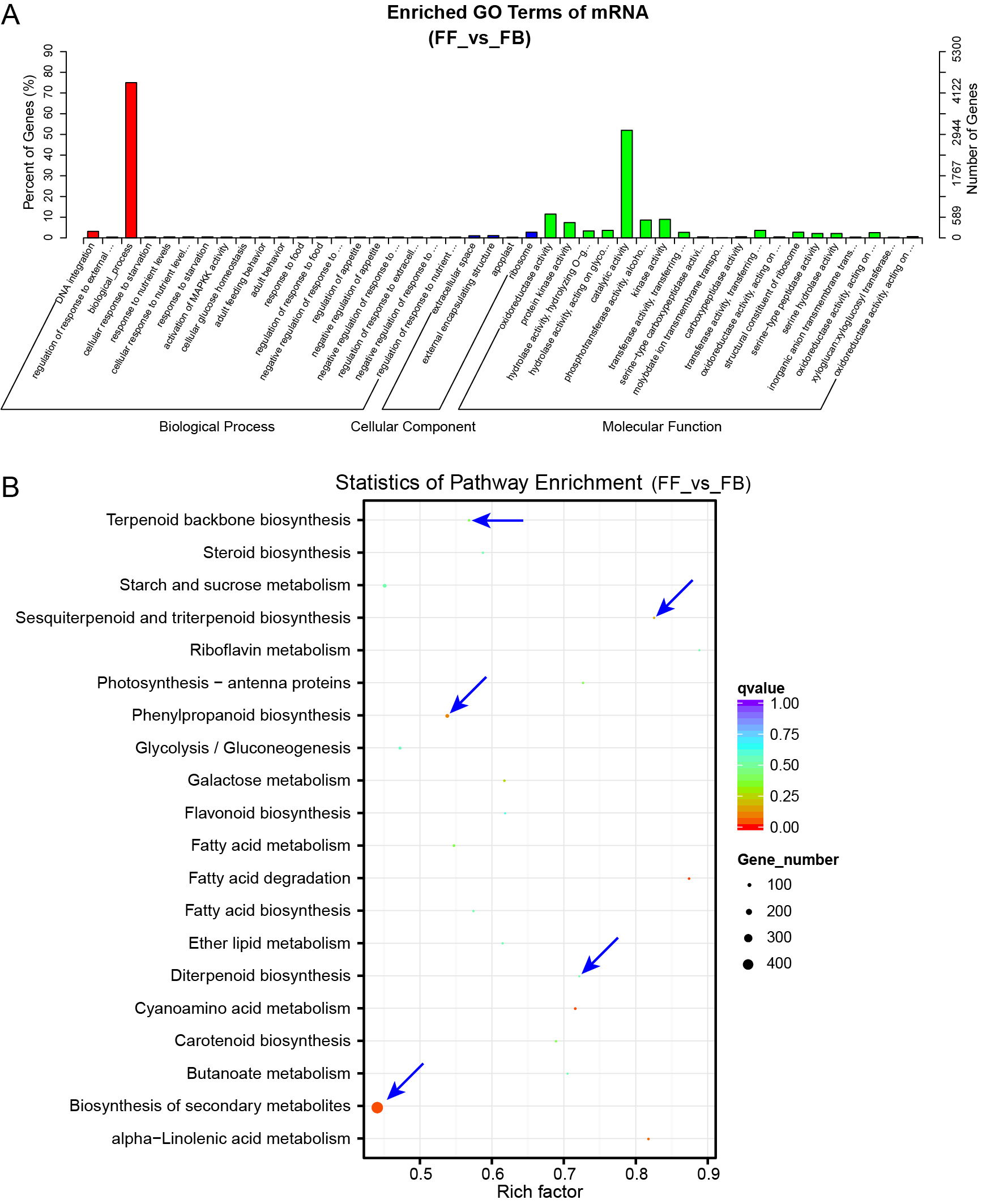

Supplement: Supplementary file 1 [file biomolecules-14-00045-s001.zip › Revised Supplementary files/Figure S1.jpg]

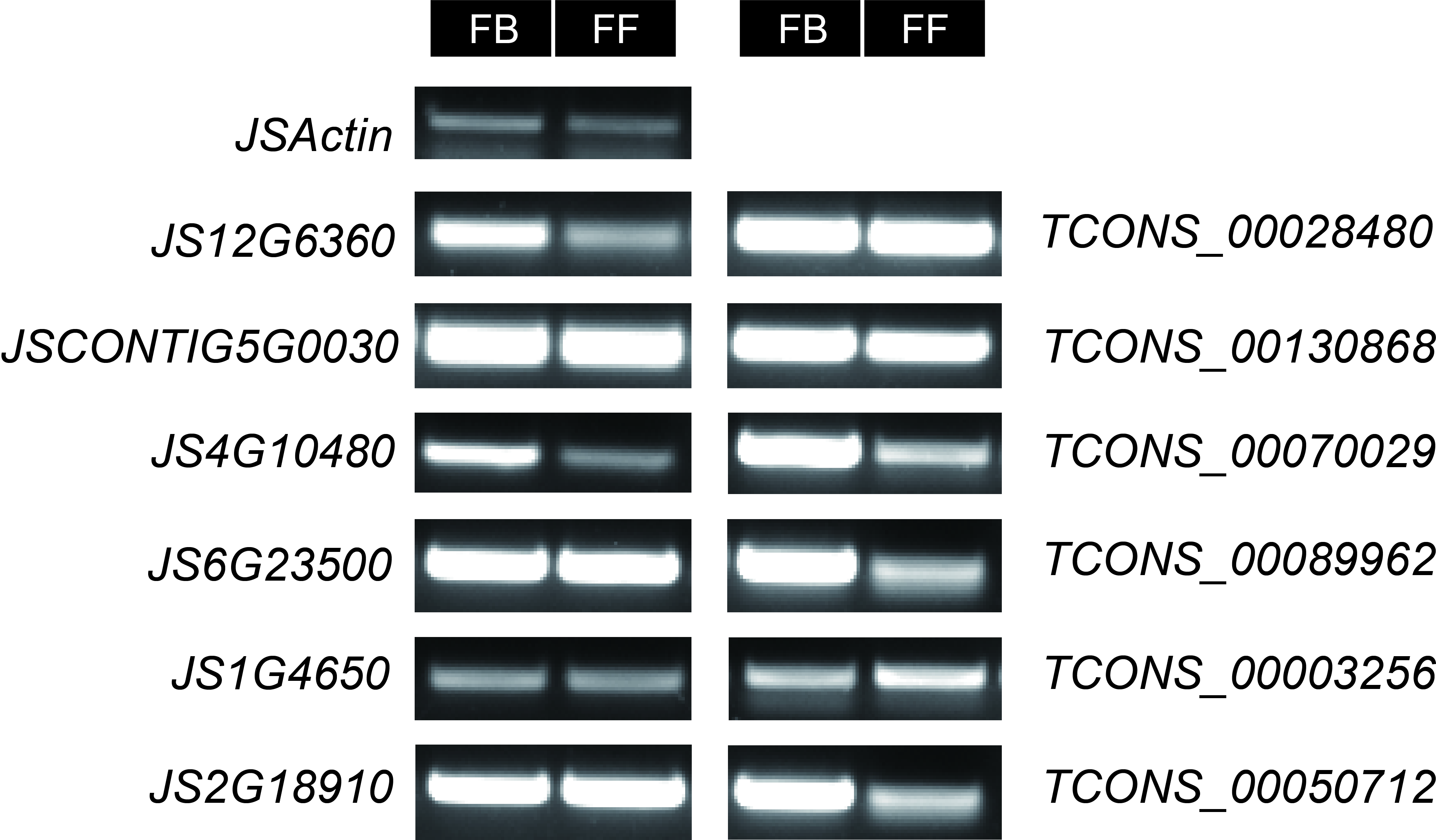

Supplement: Supplementary file 1 [file biomolecules-14-00045-s001.zip › Revised Supplementary files/Figure S2.jpg]
